# Periconceptional environment predicts leukocyte telomere length in a cross-sectional study of 7–9 year old rural Gambian children

**DOI:** 10.1038/s41598-020-66729-9

**Published:** 2020-06-15

**Authors:** Kim Maasen, Philip T. James, Andrew M. Prentice, Sophie E. Moore, Caroline H. Fall, Giriraj R. Chandak, Modupeh Betts, Matt J. Silver, Jessica L. Buxton

**Affiliations:** 1 Division of Human Nutrition, Wageningen University, Wageningen, The Netherlands; 2 Medical Research Council Unit The Gambia at the London School of Hygiene and Tropical Medicine, Atlantic Boulevard, Fajara, Banjul, The Gambia; 3 Department of Internal Medicine, Maastricht University Medical Centre, Maastricht, The Netherlands; 4 Department of Population Health, London School of Hygiene and Tropical Medicine, London, UK; 5 Department of Women and Children’s Health, King’s College London, London, UK; 6 MRC Lifecourse Epidemiology Unit, University of Southampton, Southampton General Hospital, Southampton, UK; 7 Genomic Research on Complex diseases (GRC Group), CSIR-Centre for Cellular and Molecular Biology, Hyderabad, India; 8 Division of Infection and Immunity, University College London, London, England UK; 9 Genetics and Genomic Medicine Programme, UCL Great Ormond Street Institute of Child Health, London, UK; 10 School of Life Sciences, Pharmacy and Chemistry, Kingston University, London, UK

**Keywords:** Ageing, Epidemiology, Paediatric research, Developmental biology, Medical research, Genetics research

## Abstract

Early life exposures are important predictors of adult disease risk. Although the underlying mechanisms are largely unknown, telomere maintenance may be involved. This study investigated the relationship between seasonal differences in parental exposures at time of conception and leukocyte telomere length (LTL) in their offspring. LTL was measured in two cohorts of children aged 2 yrs (N = 487) and 7–9 yrs (N = 218). The association between date of conception and LTL was examined using Fourier regression models, adjusted for age, sex, leukocyte cell composition, and other potential confounders. We observed an effect of season in the older children in all models [likelihood ratio test (LRT) χ²_2_ = 7.1, p = 0.03; fully adjusted model]. LTL was greatest in children conceived in September (in the rainy season), and smallest in those conceived in March (in the dry season), with an effect size (LTL peak–nadir) of 0.60 z-scores. No effect of season was evident in the younger children (LRT χ²_2_ = 0.87, p = 0.65). The different results obtained for the two cohorts may reflect a delayed effect of season of conception on postnatal telomere maintenance. Alternatively, they may be explained by unmeasured differences in early life exposures, or the increased telomere attrition rate during infancy.

## Introduction

The developmental origins of health and disease (DOHaD) paradigm holds that early life factors are important determinants of disease risk in adult life^1,2^. The processes involved in gamete maturation, conception and early embryogenesis appear particularly vulnerable to environmental exposures (reviewed in^3^). The biological mechanisms underlying associations between the periconceptional environment and future health remain poorly understood, although persistent epigenetic changes may play a role^4–7^. Another molecular process through which the effects of early life factors may exert enduring changes to cellular and organismal phenotypes is the maintenance of telomeres: nucleoprotein structures that protect the ends of linear chromosomes. A potential role for telomere biology in the developmental programming of adult disease is supported by preliminary evidence from both animal and human studies^8–11^.

Vertebrate telomeres consist of variable numbers of a tandem repeat sequence, (TTAGGG)_n_, bound to the shelterin protein complex^12^. In germ cells and embryonic tissues, telomere length is maintained through the addition of *de novo* telomeric DNA repeats to the chromosome ends by telomerase. However, this enzyme is generally not active in differentiated cells postnatally. Therefore, in proliferative tissues, telomeres shorten with each cell division due to the ‘end replication problem’, a process accelerated by oxidative stress^13^ and inflammation^14^, and potentially ameliorated by antioxidants in the diet and vitamin intake^15–17^.

Shorter age-adjusted leukocyte telomere length (LTL) in humans is associated with increased risk of several non-communicable diseases, notably cardiometabolic conditions^18,19^. LTL is also associated with several known risk factors for ill health, including obesity and smoking^20^ and with genetic factors^21,22^. However, the proportion of variation in adult LTL explained by these factors is relatively small compared to the wide variation in telomere length that exists in newborns^23,24^. Research into the early life determinants of telomere length implicates the prenatal environment^25–31^.

In the present study, we sought to investigate the relationship between the periconceptional environment and telomere length in children born in the rural Kiang West region of The Gambia. This region is marked by a distinct tropical seasonality, with an intense rainy season from July to September, coinciding with a period of dietary restriction (the ‘hungry’ season) but a greater availability and consumption of green and leafy vegetables lasting for several months. The dry season lasts for the remainder of the year, with February to April being peak dry season. As a result, conceptions and births in this setting occur against very different exposures to parental energy balance, diet composition, maternal nutritional status and infections^32–34^. Seasonal changes in early life in this population have been linked to differences in mortality, and to epigenetic changes in offspring^35–40^. We therefore tested the hypothesis that LTL is associated with timing of conception in children from two established Gambian population cohorts.

## Results

Details of the two cohorts studied – the Early Nutrition and Immune Development, ISRCTN49285450 (ENID) trial and the Gambian arm of the Epigenetic Mechanisms linking Pre-conceptional nutrition and Health Assessed in India and Sub-Saharan Africa, ISRCTN14266771 (EMPHASIS) study - are given in the Methods, and characteristics of the participants are summarised in Table 1. We obtained relative LTL measurements (as ‘T/S’ ratios, using monochrome multiplex quantitative PCR, see Methods for details) for 487 children in the ENID cohort (median T/S ratio 1.8, IQR 1.5–2.1) and 218 children in the EMPHASIS cohort (median T/S ratio 1.1, IQR 0.9–1.3). The decrease in median T/S ratio between the cohorts (95% CI [0.63–0.74]) is consistent with the fact that telomere length declines with age.

**Table 1:** General characteristics of the ENID and EMPHASIS study participants.

	ENID (N = 487)	EMPHASIS (N = 218)
Age (years)^a^	2.0 (2.0-2.0)	9.0 (8.6-9.2)
Sex (% male)	50	55
Maternal folate concentration (nmol/L)^a^		
Method A (Architect system)	12.8 (9.8-16.7)	—
Method B (EDTA plasma folate)	13.2 (9.3–18.9)	—
Maternal BMI^a^	20.4 (19.0–22.3)	20.8 (19.3–22.9)
Child birthweight (grams) ^b^	3023 ± 402	3069 ± 417
Maternal intervention arm^c^		
FeFol	120 (25)	—
MMN	130 (27)	—
PE	118 (24)	—
PE + MMN	119 (24)	—
UNIMMAP	—	102 (47)
Placebo	—	116 (53)
Infant intervention arm, n (%)		
LNS^c^	247 (51)	—
+ MMN	240 (49)	—

^a^Median (IQR).

^b^Mean ± SD.

^c^n (%).

BMI, Body Mass Index, FeFol, iron and folic acid; MMN, multiple micronutrient; PE, protein energy; LNS, lipid-based nutritional supplement; UNIMMAP, UNICEF/WHO/United Nations University Multiple Micronutrient Preparation.

### Association between date of conception and leukocyte telomere length

#### ENID cohort (2 year old children)

**Figure 1:**
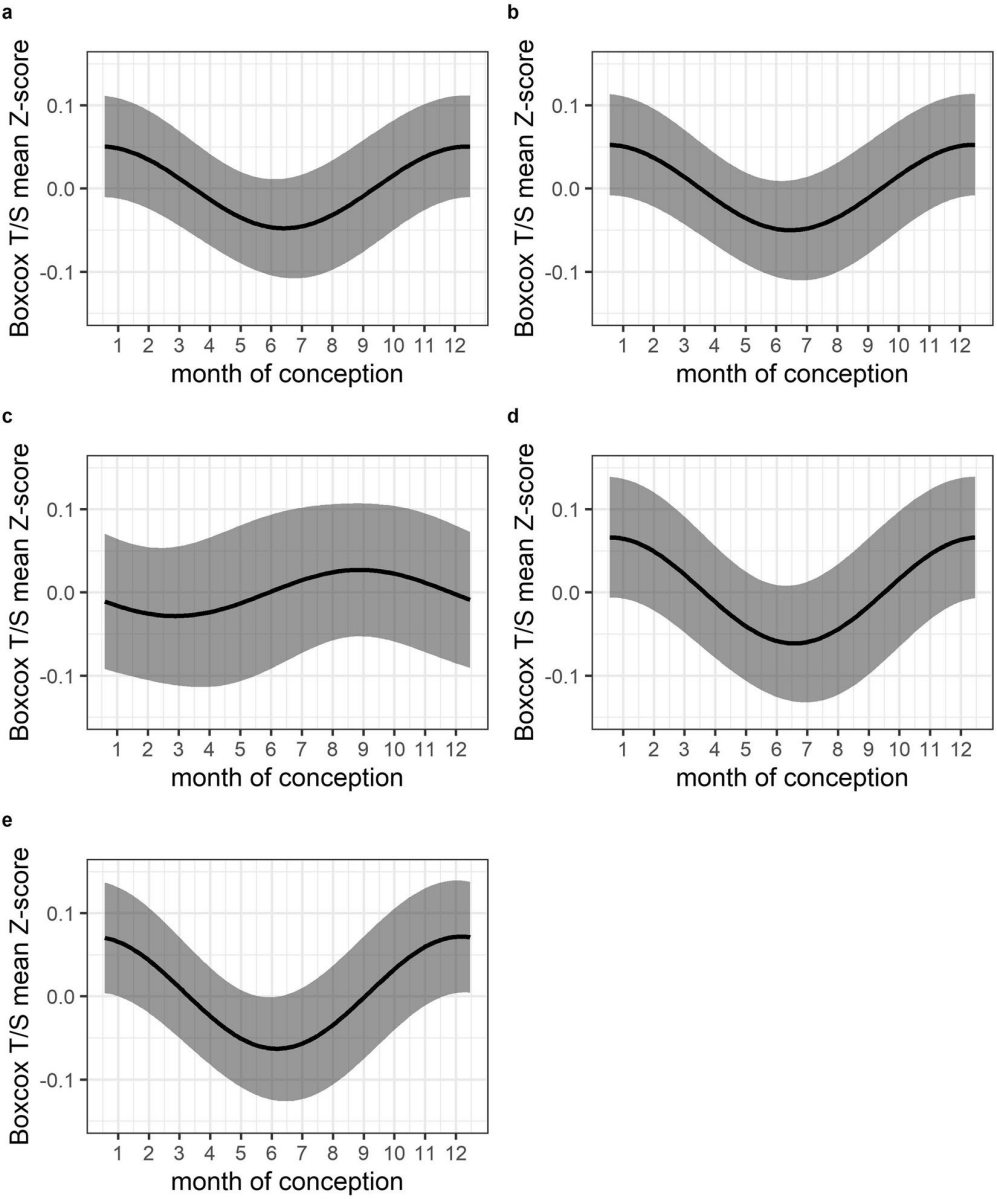
ENID cohort. Modelled associations between LTL (Boxcox transformed T/S mean z-score) and seasonality using Fourier regression. Grey shaded areas are 95% confidence intervals. (**a**) Model 1 - crude model; (**b**) Model 2 - adjusted for age and sex; (**c**) Model 3 - adjusted for age, sex and estimated cell composition; (**d**) Model 4 - adjusted for age, sex and maternal BMI; (**e**) Model 5 - adjusted for age, sex and birthweight.

In our primary analysis, we found no evidence for a seasonal effect on LTL when comparing a crude model with seasonality modelled as a single pair of Fourier terms with a baseline (intercept-only) model (likelihood ratio test (LRT) χ²_2_ = 4.24, p = 0.12; Table 2, Model 1; Fig. 1a). In subsequent secondary analyses adjusted for additional covariates (Fig. 1b-e and Supplementary Fig. S3), there was weak evidence for a seasonal effect in models adjusted for maternal body mass index (BMI) (LRT χ²_2_ = 5.48, p = 0.06; Table 2, Model 4; Fig. 1d) and birthweight (LRT χ²_2_ = 5.86, p = 0.05; Table 2, Model 5; Fig. 1e). Adjustment for estimated white cell composition had the largest effect on Fourier coefficient estimates, as indicated by a large difference in the timing of seasonal phase (Table 2, Model 3; Fig. 1c and Supplementary Table S1). Note that sample size was much reduced for this model since DNA methylation data for estimation of cellular fractions was only available for a limited number of samples (n = 204). Note also that use of more than 1 pair of Fourier terms to model seasonality did not improve model fits (data not shown).

**Table 2:** ENID cohort. Association between LTL (T/S mean z-score) and seasonality using Fourier regression models.

	N	LRT χ^2^ (2 df)	LRT p-value
Model 1 – crude **seasonality modelled with one pair of Fourier terms**^**a**^ **Baseline: intercept only**	487	4.24	0.12
Model 2 – adjusted for age and sex **1 pair Fourier terms + age + sex Baseline: age and sex only**	487	4.24	0.11
Model 3 – adjusted for age, sex and estimated white cell composition **1 pair Fourier terms + age + sex + cell composition Baseline: age + sex + cell composition only**	204	0.87	0.65
Model 4 – adjusted for age, sex and maternal BMI **1 pair Fourier terms + age + sex + maternal BMI Baseline: age + sex + maternal BMI only**	345	5.48	0.06
Model 5 – adjusted for age + sex + birthweight **1 pair Fourier terms + age + sex + birthweight Baseline: age + sex + birthweight**	395	5.86	0.05

^a^In each case, model fit is assessed by comparing the full and baseline models using a likelihood ratio test (LRT). LRT confirmed that two/three pairs of Fourier terms did not improve model fit. BMI, Body Mass Index.

#### Effect of white blood cell composition on LTL – ENID cohort

To further explore the individual effect of white blood cell composition on LTL, we compared a full model with cell composition estimates as predictors to a baseline (intercept-only) model. This indicated no cell composition effect (LRT χ²_2_ = 2.19, p = 0.82). Coefficients for individual cell proportions also indicated no independent cell type effects (p-values from 0.21–0.87).

#### EMPHASIS cohort (7–9 year old children)

**Figure 2:**
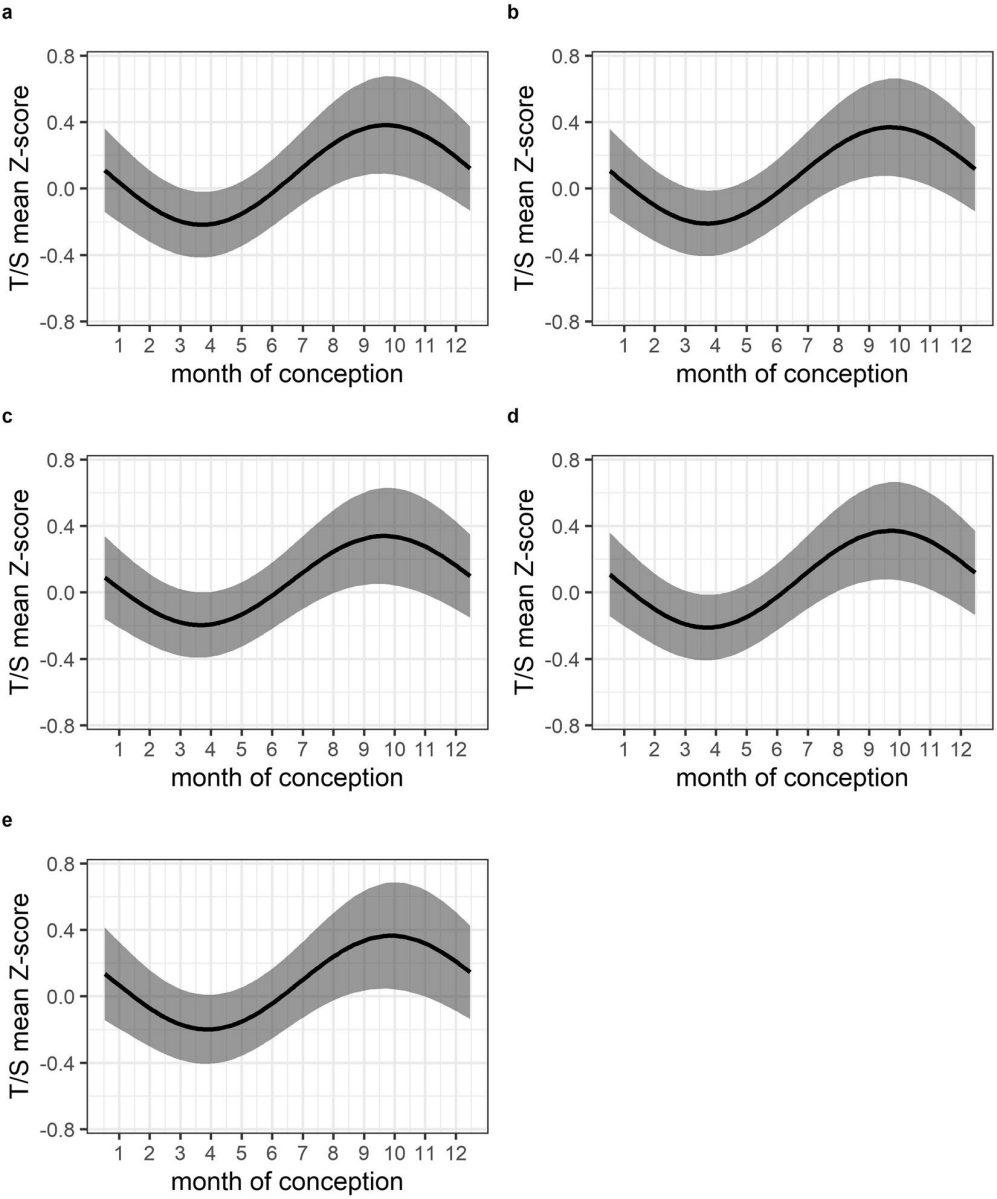
EMPHASIS cohort. Modelled associations between LTL (T/S mean z-score) and seasonality using Fourier regression. Grey shaded areas are 95% confidence intervals. (**a**) Model 1 - crude model; (**b**) Model 2 - adjusted for age and sex; (**c**) Model 3 - adjusted for age, sex and estimated cell composition; (**d**) Model 4 - adjusted for age, sex and maternal BMI; (**e**) Model 5 - adjusted for age, sex and birthweight.

For the EMPHASIS cohort, a primary crude analysis with seasonality once again modelled as a single pair of Fourier terms found evidence for a seasonal effect on LTL (LRT χ²_2_ = 8.27, p = 0.016; Table 3, Model 1; Fig. 2a). The modelled T/S ratio mean reached a maximum for individuals conceived in September (rainy season), and a minimum in March (dry season), with an effect size (amplitude = T/S mean z-score maximum – minimum) of 0.60 SD. (Fig. 2a). Evidence for a seasonality effect was also evident in models adjusted for child age and sex (χ²_2=_7.89, p = 0.019, amplitude=0.56; Table 3, Model 2; Fig. 2b) and in further models additionally adjusted for white cell composition, maternal BMI, birthweight and maternal supplementation (Table 3, Models 3–5; Fig. 2c–e; Supplementary Table S2, Model 6; and Supplementary Fig. S4). In every case the temporal nature (phase) of the seasonal association did not change, with no evidence for a change in estimated Fourier regression coefficients, and modelled T/S mean maxima in September, and minima in March (Fig. 2 and Supplementary Table S2). Use of more than 1 pair of Fourier terms to model seasonality did not improve model fits (data not shown).

**Table 3:** EMPHASIS cohort. Association between LTL (T/S mean z-score) and seasonality.

	N	LRT χ^2^ (2 df)	LRT p-value
Model 1 – crude **seasonality modelled with one pair of Fourier terms**^**a**^ **Baseline: intercept only**	218	8.27	**0.016**
Model 2 – adjusted for age and sex **1 pair Fourier terms + age + sex Baseline: age and sex only**	218	7.89	**0.019**
Model 3 – adjusted for age, sex and estimated white cell composition **1 pair Fourier terms + age + sex + cell composition Baseline: age + sex + cell composition only**	214	7.10	**0.029**
Model 4 – adjusted for age, sex and maternal BMI **1 pair Fourier terms + age + sex + maternal BMI Baseline: age + sex + maternal BMI only**	218	7.96	**0.019**
Model 5 – adjusted for age + sex + birthweight **1 pair Fourier terms + age + sex + birthweight Baseline: age + sex + birthweight**	200	6.51	**0.039**

^a^In each case, model fit is assessed by comparing the full and baseline models using a likelihood ratio test (LRT). LRT confirmed that two/three pairs of Fourier terms did not improve model fit. BMI, Body Mass Index.

### Other early-life predictors of LTL in ENID and EMPHASIS cohorts

For both cohorts, we additionally explored the individual effect of maternal BMI, birthweight, maternal folate (ENID cohort only), maternal supplementation and infant supplementation (ENID only) on LTL. After accounting for potential seasonality effects, we found no evidence of associations with LTL in either of the cohorts (Supplementary Table S3 and Supplementary Figures S2 and S3).

## Discussion

This study explored whether the periconceptional environment is associated with telomere length in children living in a rural area of The Gambia. We show that timing of conception in this highly seasonal environment predicts LTL in children aged 7–9 years. Children in this cohort (EMPHASIS) who were conceived mid-dry season (March) have the shortest mean LTL, while those conceived towards the end of the rainy season (September) have the longest mean LTL. This effect is not explained by differences in white blood cell composition at the time of blood collection, maternal periconceptional BMI, child birthweight, or supplementation of mothers or infants. Interestingly, we found no evidence for an association between timing of conception and LTL in children aged 2 years from the ENID cohort despite a greater sample size.

Our main finding, that children conceived mid-dry season have shorter telomeres at age 7–9 than their peers conceived mid-rainy season, is the first reported evidence for potential early life influences on telomere length in Gambian children. Our previous preliminary study of young men (18–23 y) in this population found that individuals born in the rainy season tended to have shorter CD8+lymphocyte telomeres than those born in the dry season^11^. Mean LTL at any given age is determined both by initial LTL at birth and its attrition over time. Taken together, our studies indicate that season of conception/birth is associated with TL in young adults and older children. This suggests that the effect of periconceptional environment on telomere maintenance processes, if it exists, may be delayed until mid-childhood onwards.

Accelerated telomere shortening in leukocytes during childhood may be due to a greater burden of infectious disease, as demonstrated by studies reporting substantial telomere shortening after malaria infection^41–43^. Furthermore, a study conducted in the Philippines found an association between higher rates of early-life infections and shorter adult blood telomere length^44^. It is possible that our findings may also reflect a long-term effect of differences in infectious disease burden during infancy, although this would not explain the lack of an association between LTL and season of conception in the younger cohort. In addition, such an effect would be at least partly mitigated by exclusive breastfeeding, rates of which are high in this population^45^.

**Figure 3:**
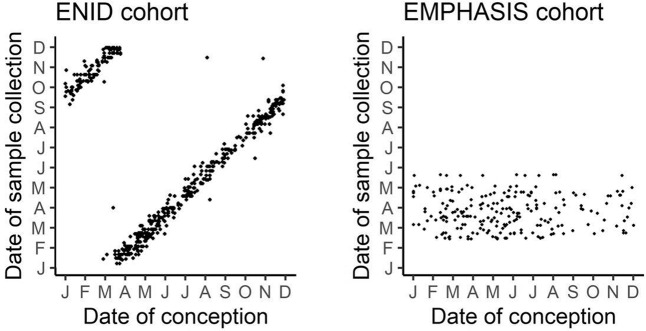
Date of conception versus date of collection ENID and EMPHASIS cohorts. These are correlated for the ENID cohort since the majority of samples are collected when the children are 2 years old. (J-D, Jan-Dec).

Our finding that timing of conception predicts telomere length in mid-childhood, but not infancy, in this population could also potentially be due to elements of our study design. The two cohorts differed in timing of sample collection. In the EMPHASIS cohort all blood samples were collected in a 4-month window spanning the middle of the dry season, whereas in the ENID cohort samples were collected throughout the year when the child reached the age of 2 years. Therefore, in the ENID cohort date of conception is very strongly correlated with date of sample collection (Fig. 3), so any effect of conception timing maybe confounded by an effect related to timing of sample collection. Previous research carried out in Costa Rica, which also has distinct rainy and dry seasons, found that season of blood collection is associated with adult LTL and that this may mask the effects of other factors^46,47^. These findings could reflect seasonal differences in leukocyte cell composition, although we found no evidence for this in our sample. Alternatively, the lack of association between LTL and season of conception in the younger cohort may be partly or wholly explained by the faster telomere attrition rate known to exist in infancy, compared to that in older children and adults^48^. Variation in the timing of this process in each child could have obscured an association between LTL and earlier effects in our younger cohort.

Previous studies report that higher maternal pre-pregnancy BMI and lower maternal folate concentrations during pregnancy are associated with shorter newborn telomere length^49,50^. Low birthweight is also associated with shorter telomere length in 5-year-old children^51^. We found no evidence of confounding of our main observations by these or other *in utero* exposures. We also found no evidence of independent associations of these early-life predictors with LTL, although there was weak evidence of an association between increased periconceptional maternal BMI and shorter LTL prior to adjustment for date of conception.

Our study has several strengths. First, the seasonal ‘experiment of nature’ setting of this well-documented population allowed us to examine the effect of distinct differences in periconceptional environment. Second, the availability of relatively large numbers of samples from two established cohorts enabled us to explore determinants of telomere length at two stages in early life. Third, the availability of anthropometric and biochemical measurements facilitated adjustment for multiple potential confounders and the exploration of potential mediators. A limitation of our study is that we measured relative, rather than absolute telomere length, so were not able to estimate the effect of timing of conception in base-pairs.

In summary, we show that timing of conception predicts leukocyte telomere length in 7–9 year old children, with the shortest LTL for children conceived mid-dry season and the longest LTL for those conceived towards the end of the rainy season. This association was not explained by other (pre)pregnancy or early-life factors, such as maternal periconceptional BMI, child birthweight, maternal and infant supplementation, or maternal folate concentration. To the best of our knowledge, this is the first study to report an association between date of conception and telomere length in children in a West-African setting. Most studies into the determinants and consequences of short telomeres have so far been conducted in high income countries, so their relevance to healthy ageing in nutritionally deprived individuals who face a high burden of infectious disease is unknown. The identification of novel factors associated with telomere length in Gambian children may help elucidate potential mechanisms through which prenatal and early-life exposures affect adult health in this and other populations. Future longitudinal studies would provide greater insight into telomere attrition rate throughout life, and help identify determinants of healthy development and ageing.

## Methods

### Study population

DNA for LTL measurements was prepared using stored blood samples from two established population cohorts from the Kiang West region of The Gambia. This population, consisting of approximately 15,000 individuals, depends predominantly on subsistence farming, and has been described in detail previously^52^.

#### ENID cohort

The ENID trial was a 2 × 2 × 2 factorial randomised, partially blinded trial in which pregnant women (<20 weeks gestation) and their infants (from 6 to 18 months of age) received nutritional supplementation^53^ (See Supplementary Methods for details). Venous blood samples were collected from children at when they turned 2 years old, between 2012–2016 (see Fig. 3). In total, blood DNA samples were available from 581 children. Additionally, venous blood from mothers was collected at booking (around 12 weeks gestation) after an overnight fast.

#### EMPHASIS cohort

The Gambian arm of the EMPHASIS study followed up children of mothers who were enrolled in the Peri-conceptional Multiple Micronutrient Supplementation Trial (PMMST)^54,55^ (See Supplementary Methods for details). The present study utilised DNA collected from venous blood samples from the EMPHASIS children at age 7-9 yrs. All blood samples were collected mid-dry season between February and May 2016 (see Fig. 3). In total, blood DNA samples were available from 287 children.

Further details on study participants from both cohorts are given in Table 1.

#### Ethics statement

Both trials were approved by the joint Gambia Government/MRC Unit The Gambia Ethics Committee and informed consent was obtained from all participants (ENID: SCC1126v2; EMPHASIS: SCC1441v2; LTL study: L2016.03). All methods were performed in accordance with the relevant guidelines and regulations.

### Assessment of date of conception

Estimated date of conception was obtained using gestational age data, estimated by ultrasound at the first (‘booking’) clinic visit.

### Blood collection and DNA extraction

Venous blood was collected into EDTA tubes and stored at −80 °C. For the ENID study, genomic DNA was extracted from peripheral blood using the salting-out method^56^ for the majority of samples (n = 450 - of total analysed samples after exclusions) and the Nucleon kit (Nucleon Bacc2 DNA extraction kits, Scientific Laboratory Supplies Ltd, Nottingham, UK) for the remainder of the samples (n = 37). Mean LTL did not differ between the two extraction methods (p = 0.75). For the EMPHASIS samples, genomic DNA was extracted using the Qiagen DNA Blood Midi Kit (Qiagen, Crawley, UK). All DNA samples were resuspended or eluted in RT-PCR water, and dilutions of 10 ng/μl were prepared using RT-PCR Grade Water (Thermo Fisher Scientific) approximately 2-6 weeks before carrying out telomere measurements. Additionally, in the ENID study whole venous blood from mothers was collected after an overnight fast for maternal folate measurements.

### Telomere length measurement

Mean relative LTL was measured using monochrome multiplex quantitative PCR (MMqPCR) in genomic DNA samples with minor modifications^57^ (see Supplementary Methods).

After amplification and data collection, the CFX manager software (Bio-Rad Laboratories) was used to generate standard curves from the reference DNA dilutions, one for the telomere amplicon (T) and one for the single-copy gene amplicon (S). A “T/S ratio” was then calculated for each DNA sample - a relative measure of the amplification of the telomeric DNA sequence compared to that of the single copy gene (albumin gene – see supplementary methods for primer sequences).

After excluding samples that failed to amplify and those with a coefficient of variation of >15% between duplicates, the mean coefficient of variation for the T/S measurements was 5.5% for the ENID samples and 4.7% for the EMPHASIS samples. Inter-assay coefficients of variation, calculated using the reference sample and control samples common to each plate, were 8.6% and 16.9% for ENID and EMPHASIS samples respectively. For the three control samples, aged 2, 26, and 72 years, mean T/S ratios were 1.90, 1.54 and 1.09 respectively, showing an expected decrease in T/S ratio over age.

Sufficient DNA and reagents were available for telomere measurements in a total of 533 children from the ENID cohort and 287 children from the EMPHASIS cohort. After exclusions, T/S measurements were available for statistical analyses for 487 children (91%) from the ENID cohort and 218 children (76%) from the EMPHASIS cohort (see Supplementary Figs. S1 and S2 for details).

### Statistical methods

All LTL measurements (T/S ratio) were converted to a per plate z-score, in order to account for a batch effect observed in measurements obtained for both cohorts (ANOVA, p < 0.001). The main outcome variable (LTL z-score) was normally distributed (by visual inspection of histogram) in the EMPHASIS cohort, and in the ENID cohort after Boxcox transformation of LTL z-scores. Skewed predictor variables (maternal BMI and maternal folate status) were log-transformed, and fitted models had normally distributed residuals, with no evidence of homoscedasticity or multicollinearity.

The association between date of conception and LTL was examined in multiple linear regression models using Fourier terms to capture seasonal variation^58,59^. Fourier regression-based approaches allow for decomposition of any periodic function into a linear combination of simple oscillating functions (sines and cosines) parametrized by coefficients (the Fourier coefficients). This approach allows the modelling of cyclical variation with no prior assumptions about the frequency, phase or amplitude of any seasonal effects.

In our analysis, seasonality was modelled using pairs of Fourier terms (predictors), with the number of pairs determined using a likelihood ratio test (LRT). We began with a crude primary model that included seasonality modelled as Fourier terms only. We subsequently performed sensitivity analyses by separately adding potential confounders of the relationship between seasonality and LTL. The confounders that we considered were the child’s age, sex, white blood cell composition (estimated from DNA methylation data using the Houseman method^60^), maternal periconceptional BMI, child birthweight, maternal and infant supplementation, and maternal folate concentration (in ENID cohort only) (see Supplementary Methods). Missing data for some of these variables meant that sample numbers varied between the models considered.

Lastly, to explore the individual effect of other early-life exposures on LTL, we performed multiple linear regression analyses with maternal BMI, birthweight, supplementation status, and maternal folate as predictors. All models were adjusted for age and sex of children. Predictors with a known seasonal variation (maternal BMI, birthweight, and maternal folate) were additionally adjusted for seasonality using one pair of Fourier terms. All analyses were performed using R version 3.3.0 (www.cran.r-project.org).

## Supplementary information

Supplementary Information.

Supplementary Information2.

## Data Availability

The datasets generated during and/or analysed during the current study are available from the corresponding authors on reasonable request.
