# Supplementary material for: Periconceptional environment predicts leukocyte telomere length in a cross-sectional study of 7–9 year old rural Gambian children
Source: Sci Rep. 2020 Jun 15;10:9675. doi: 10.1038/s41598-020-66729-9 (PMC7295801; doi:10.1038/s41598-020-66729-9)
Supplement: Supplementary file 1 — Supplementary Information. [file 41598_2020_66729_MOESM1_ESM.pdf]

Supplementary file - Online Supplementary Material

Periconceptional environment predicts leukocyte telomere length in a cross-sectional study of 7-9 year old rural Gambian children

Kim Maasen, Philip T James, Andrew M Prentice, Sophie E Moore, Caroline H Fall, Giriraj R Chandak, Modupeh Betts, Matt J Silver[#] and Jessica L Buxton[#*]*

*[#] Joint senior authors*Corresponding author*

Supplementary tables

Supplementary table S1 – Seasonality Fourier term regression coefficients for ENID cohort regression models described in Table 2 [1]

	**N**	**Beta coefficient**	**SE**	**Linear regression p-value**
**Model 1 (crude seasonality only)**	487			
Sin (date of conception)		0.005	0.026	0.853
Cos (date of conception)		0.052	0.025	**0.040**
**Model 2 (model 1 + age and sex)**	487			
Sin (date of conception)		0.005	0.026	0.835
Cos (date of conception)		0.058	0.026	**0.023**
**Model 3 (model 2 + cell composition)**	204			
Sin (date of conception)		-0.028	0.036	0.440
Cos (date of conception)		-0.013	0.038	0.733
**Model 4 (model 2 + maternal BMI)**	345			
Sin (date of conception)		0.008	0.033	0.817
Cos (date of conception)		0.077	0.030	**0.011**
**Model 5 (model 2 + birthweight)**	395			

Sin (date of conception)		-0.004	0.029	0.877
Cos (date of conception)		0.071	0.028	**0.012**
**Model 6 (model 2 + supplementation group – 8 groups)**	487			
Sin (date of conception)		0.008	0.026	0.763
Cos (date of conception)		0.057	0.026	**0.027**
**Model 7 (model 2 + maternal supplementation group – 4 groups)**	487			
Sin (date of conception)		-0.004	0.026	0.878
Cos (date of conception)		0.058	0.026	**0.026**
**Model 8 (model 2 + infant supplementation group – 4 groups)**	487			
Sin (date of conception)		0.007	0.026	0.798
Cos (date of conception)		0.059	0.026	**0.021**
**Model 9 (model 2 + maternal folate concentration)**	378			
Sin (date of conception)		0.005	0.030	0.871
Cos (date of conception)		0.059	0.030	0.055

[1] BMI, Body Mass Index; SE, standard error.

Supplementary table S2 – Seasonality Fourier term regression coefficients for EMPHASIS cohort regression models described in Table 3 [1]

	N	Beta coefficient	SE	Linear regression p-value
**Model 1 (crude seasonality only)**	218			
Sin (date of conception)		-0.30	0.10	**0.004**
Cos (date of conception)		0.03	0.09	0.72
**Model 2 (model 1 + age and sex)**	218			
Sin (date of conception)		-0.30	0.10	**0.004**
Cos (date of conception)		0.03	0.09	0.72
**Model 2a (model 2 + cell composition)**	214			
Sin (date of conception)		-0.28	0.11	**0.01**
Cos (date of conception)		0.02	0.09	0.80
**Model 2b (model 2 + maternal BMI)**	218			
Sin (date of conception)		-0.30	0.11	**0.006**
Cos (date of conception)		0.03	0.09	0.72
**Model 2c (model 2 + birthweight)**	200			
Sin (date of conception)		-0.28	0.11	**0.01**

Cos (date of conception)		0.06	0.10	0.55
**Model 2d (model 2 + maternal supplementation group)**	218			
Sin (date of conception)		-0.29	0.11	**0.006**
Cos (date of conception)		0.03	0.09	0.73

[1] BMI, Body Mass Index; SE, standard error.

Supplementary table S3 – Multiple linear regression of early life predictors on LTL for samples from the ENID and EMPHASIS cohorts[1]

	N	Beta coefficient	SE	Linear regression p-value
**ENID cohort**				
Maternal BMI	345	-0.25	0.15	0.08
Maternal BMI adjusted for seasonality[2]	345	-0.21	0.15	0.15
Birthweight	395	-0.04	0.05	0.42
Birthweight adjusted for seasonality[2]	395	-0.04	0.05	0.38
Supplementation group – 8 groups	487			
2		0.08	0.07	0.26
3		0.11	0.07	0.14
4		0.05	0.07	0.50
5		0.01	0.07	0.89
6		0.12	0.07	0.11
7		0.11	0.07	0.15
8		0.05	0.07	0.50

Maternal supplementation group – 4 groups	487			
2		0.05	0.05	0.36
3		0.02	0.05	0.66
4		-0.02	0.05	0.64
Infant supplementation group – 2 groups	487	0.03	0.04	0.40
Maternal folate concentration	378	-0.03	0.05	0.46
Maternal folate concentration + Fourier terms seasonality[2]	378	-0.008	0.05	0.87
**EMPHASIS cohort**				
Maternal BMI	218	-0.07	0.43	0.87
Maternal BMI adjusted for seasonality	218	-0.13	0.42	0.76
Birthweight	200	0.0001	0.0002	0.47
Birthweight adjusted for seasonality	200	0.0002	0.0002	0.37
Maternal supplementation group	218	-0.02	0.14	0.87

[1] All tests, multiple regression adjusted for age and sex

[2] Model includes one pair of Fourier terms to adjust for seasonality

BMI, Body Mass Index; SE, standard error.

Supplementary figures

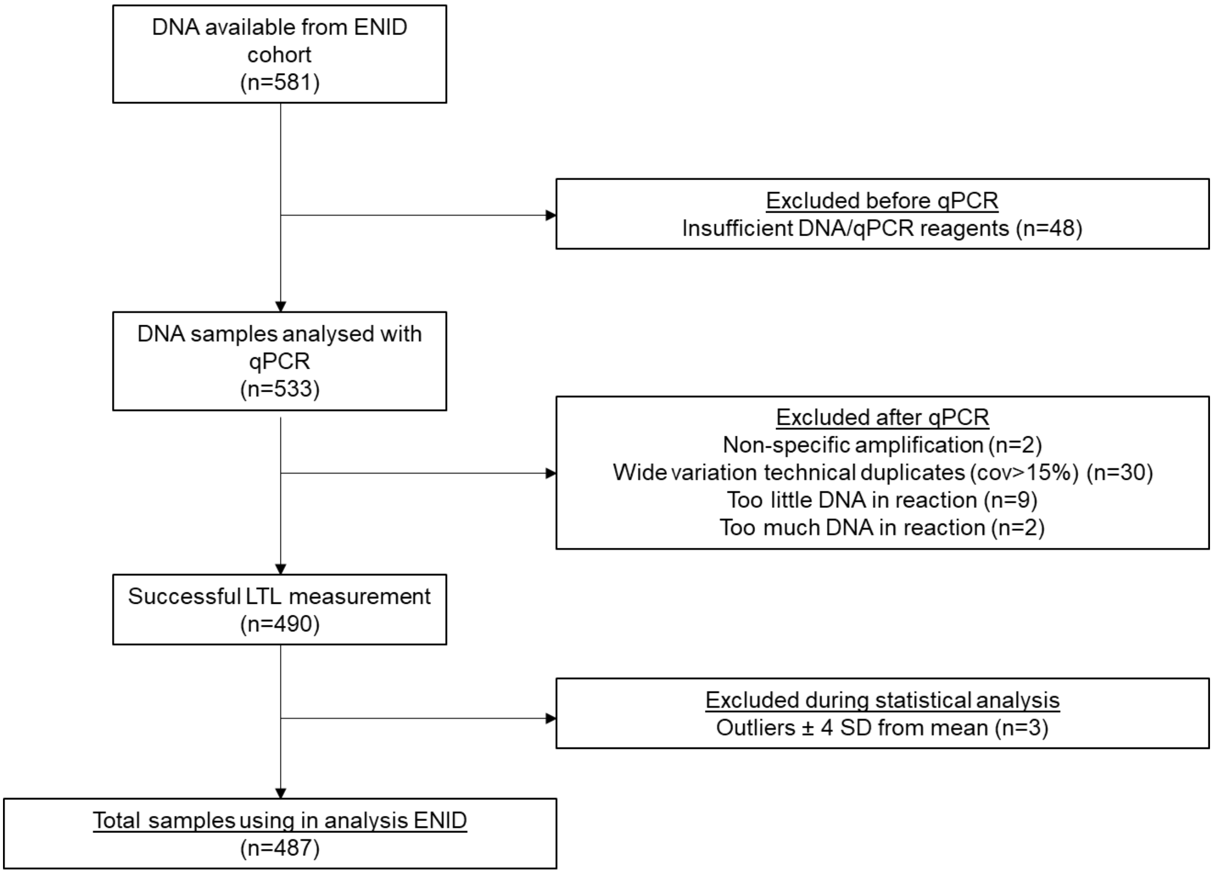

Supplementary figure S1 – Flowchart ENID samples

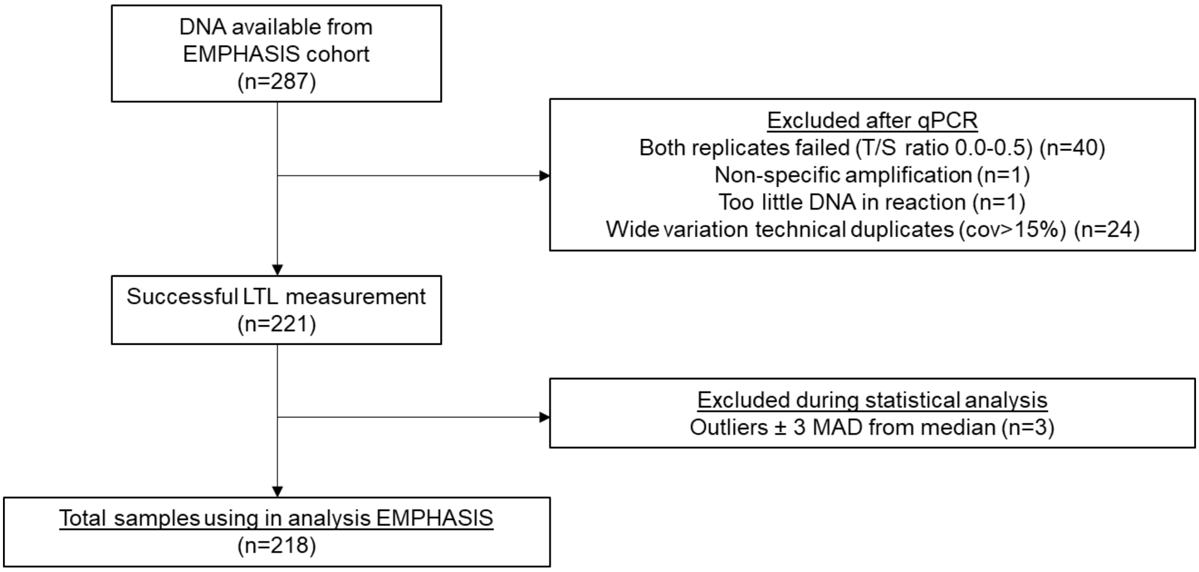

Supplementary figure S2 – Flowchart EMPHASIS samples

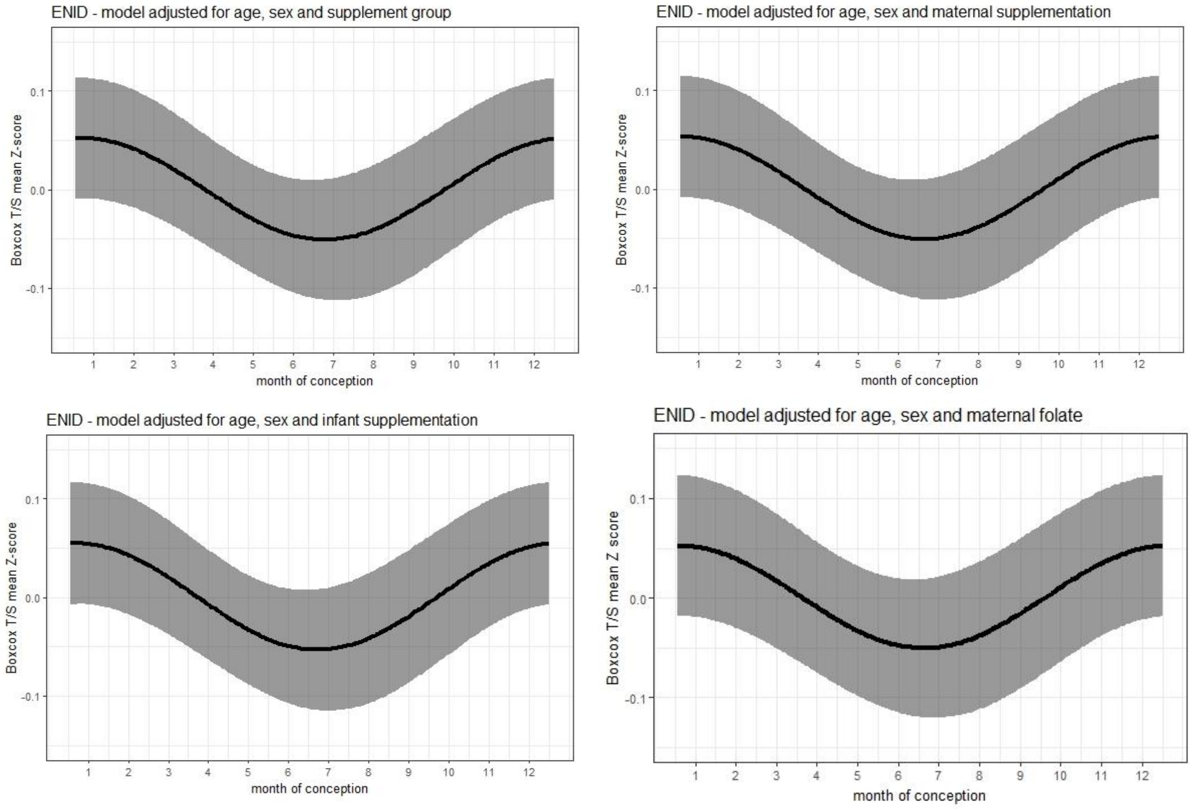

Supplementary figure S3 – ENID cohort. Modelled associations between LTL (Boxcox transformed T/S mean z-score) and seasonality using Fourier regression. Models adjusted for supplement group and maternal folate. Grey shaded areas are 95% confidence intervals.

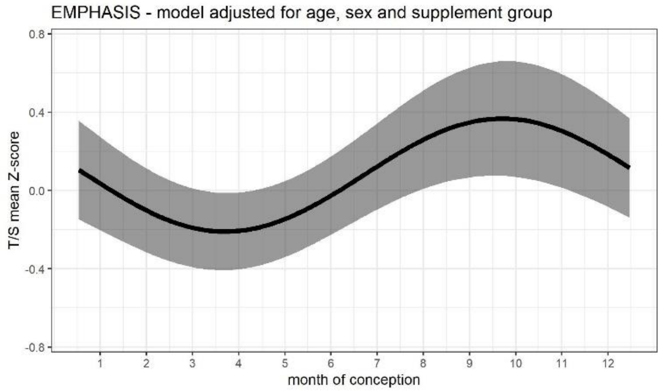

Supplementary Figure S4 - EMPHASIS cohort. Modelled associations between LTL (T/S mean z-score) and seasonality using Fourier regression. Model adjusted for supplement group. Grey shaded areas are 95% confidence intervals.

Supplementary methods

Supplementation details ENID cohort

Pregnant women were randomized to four intervention groups: (1) Iron-folate (FeFol=standard care), 2) multiple micronutrients (MMN), 3) protein-energy (PE) + FeFol and 4) PE + MMN. All four groups received the same levels of daily iron and folate supplementation (60 mg iron and 400 μg folate). From six months of age, infants were further randomised to a lipid-based nutritional supplement with or without additional MMNs. The trial took place from April 2010 to February 2015.

Supplementation details EMPHASIS cohort

The Gambian arm of the EMPHASIS study followed up children of mothers who were enrolled in the Peri-conceptional Multiple Micronutrient Supplementation Trial (PMMST). In PMMST, prepregnant women received either the UNICEF/WHO/United Nations University multiple micronutrient preparation (UNIMMAP) or a placebo until pregnancy was confirmed (median time with supplementation 24.1 weeks). Following a positive pregnancy test, the intervention was stopped and replaced with 60 mg elemental iron and 250 μg folic acid daily, in accordance with Gambian national antenatal policy. The PMMST trial took place between March 2006 and June 2008.

Leukocyte telomere length measurements

Duplicate reactions were carried out in a total volume of 25 μl, using 20ng of template DNA, with final concentrations of 1× iQ Sybr Green supermix (Bio-Rad Laboratories, Hemel Hempstead, UK), 900 nM of telg and telc primers, and 500 nM of single-copy gene primers (albUgcr1 and albDgcr1; for primer sequences see end of document). Reactions were carried

out in clear 96-well PCR plates (HSL-9901, Bio-Rad Laboratories) on a CFX96 real-time PCR detection system (Bio-Rad Laboratories). Five serial dilutions of the reference DNA sample (leukocyte DNA from a female aged 28) spanning 1.6-126 ng (for ENID) or 2.5-15 ng (for EMPHASIS) were run in triplicate on each plate, in addition to one control sample of reference DNA. For the measurements of the EMPHASIS cohort, DNA from three control samples aged 2, 26 and 72 years were also added, which were used in addition to the reference DNA control sample to assess inter-assay variation.

Covariates used for adjustment

Gestational age was estimated by ultrasound at the first (‘booking’) clinic visit, from which an estimated date of conception was obtained.

White blood cell composition proportion estimates for CD8T, CD4T, NK (Natural Killer), B-cells, Monocytes, Granulocytes (for ENID cohort only), Eosinophils and Neutrophils (both for EMPHASIS cohort only) were derived from DNA methylation data [1] using Houseman’s method [2], were available for 214 children from the EMPHASIS cohort, and for 204 children from ENID. Houseman’s method uses DNA methylation signatures of each of the principal immune components of whole blood. White blood cell composition estimates were used directly as predictors in regression modelling, but in each case excluding the proportion of one of the cell types (granulocytes in the ENID cohort and NK cells in the EMPHASIS cohort) to improve model stability, since cell proportions sum to 1.

The ENID cohort had four maternal supplementation groups and two infant supplementation group. The EMPHASIS cohort had two maternal supplementation groups (see supplementation details cohorts). In the ENID cohort, maternal periconceptional BMI was available for 360 samples. Maternal pre-pregnancy BMI was available for all samples in the EMPHASIS cohort. Child birthweight data was

available for n=410 samples in the ENID cohort and n=200 samples in the EMPHASIS cohort. Refer to study protocol papers for further details [3-5].

Maternal folate concentration (nmol/L) was available in the ENID cohort only, using two different measurement methods. In 350 samples (of which 280 samples had both folate and LTL data available) EDTA plasma on an Architect system at the University of British Columbia was used to measure plasma folate concentrations. In 368 samples (of which 221 samples had both folate and LTL data available) a plasma folate method at EWL Cambridge was used to measure folate concentration. Folate was measured in plasma samples because of superior assay precision of plasma versus red cell folate [6]. Maternal folate status was measured at booking (around 12 weeks gestation), and thereafter back-extrapolated to conception and adjusted for gestational age [7]. Pearson's correlation, Bland-Altman plot and paired t-test were used to confirm that back-extrapolated folate concentrations from both laboratory methods showed good accordance (Pearson's correlation $r=0.87$, $n=113$, $p<0.001$; paired t-test mean of differences=-0.70, $p=0.08$). Where both measures were available, maternal folate status values were combined by standardizing all values and taking the mean. Where only one measure was available this was standardised and used for analysis. After combining these values, 388 samples with LTL measurements had maternal folate data available.

Primer sequences albumin primers

albUgcr1 CGGCGGCGGGCGGCGCGGGCTGGGCGGAAACGCTGCGCAGAATCCTTG

albDgcr1 GCCCGGCCCGCCGCGCCCGTCCCGCCGCTGAAAAGTACGGTCGCCTG

From: http://www.protocol-online.org/forums/index.php?app=forums&module=forums&section=printtopic&client=printer&f=1&t=14233. Adjusted ALB primers from Cawthon, 2009.

1. Van Baak, T. E. et al. Epigenetic supersimilarity of monozygotic twin pairs. *Genome Biol.* **19,** 2 (2018).
2. Houseman, E. A. et al. DNA methylation arrays as surrogate measures of cell mixture distribution. *BMC Bioinformatics*. **13,** 86 (2012).
3. Chandak, G.R. et al. Protocol for the EMPHASIS study; epigenetic mechanisms linking maternal pre-conceptional nutrition and children's health in India and Sub-Saharan Africa. *BMC Nutrition.* **3,** 81 (2017).
4. Moore, S.E., Fulford A. J., Darboe M. K., Jobarteh M. L., Jarjou L. M. & Prentice A. M. A randomized trial to investigate the effects of pre-natal and infant nutritional supplementation on infant immune development in rural Gambia: the ENID trial: Early Nutrition and Immune Development. *BMC Pregnancy Childbirth*. **12**, 107 (2012).
5. Owens, S. et al. Periconceptional multiple-micronutrient supplementation and placental function in rural Gambian women: a double-blind, randomized, placebo-controlled trial. *Am. J. Clin. Nutr.* **102,** 1450-1459 (2015).
6. Entringer, S. et al. Maternal Folate Concentration in Early Pregnancy and Newborn Telomere Length. *Ann. Nutr. Metab.* **66,** 202-208 (2015).
7. Dominguez-Salas, P. et al. Maternal nutrition at conception modulates DNA methylation of human metastable epialleles. *Nat. Commun.* **5,** 3746 (2014).
