# Supplementary material for: Periconceptional environment predicts leukocyte telomere length in a cross-sectional study of 7–9 year old rural Gambian children
Source: Sci Rep. 2020 Jun 15;10:9675. doi: 10.1038/s41598-020-66729-9 (PMC7295801; doi:10.1038/s41598-020-66729-9)
Supplement: Supplementary file 2 — Supplementary Information2. [file 41598_2020_66729_MOESM2_ESM.docx]

**Supplementary files**

Supplementary file - Online supplementary material. **Supplementary tables:** Supplementary table S1 – Seasonality Fourier term regression coefficients for ENID cohort regression models described in Table 2; Supplementary table S2 – Seasonality Fourier term regression coefficients for EMPHASIS cohort regression models described in Table 3; Supplementary table S3 – Multiple linear regression of early life predictors on LTL for samples from the ENID and EMPHASIS cohorts. **Supplementary figures:** Supplementary figure S1 – Flowchart ENID samples; Supplementary figure S2 – Flowchart EMPHASIS samples. Supplementary figure S3 - Modelled associations between LTL and supplementation, maternal folate in ENID cohort; Supplementary figure S4 – Modelled association between LTL and supplementation in EMPHASIS in cohort. **Supplementary methods:** Supplementation details ENID cohort; Supplementation details EMPHASIS cohort; Leukocyte telomere length measurements; Covariates used for adjustment; Primer sequences albumin gene primers.
